# The DECON pilot project investigates predictive markers for successful bariatric surgery

**DOI:** 10.1038/s41598-023-40452-7

**Published:** 2023-08-17

**Authors:** Gabriel Seifert, Luca Fagnocchi, Michael Edozie, Stephan Herrmann, Hannah Baumann, Ilaria Panzeri, Stephanie Mewes, David Aicher, Mira Runkel, Claudia Lässle, Jodok Fink, Goran Marjanovic, Stephan Fichtner-Feigl, J. Andrew Pospisilik

**Affiliations:** 1 Department of General and Visceral Surgery, University Medical Center Freiburg, University of Freiburg, Freiburg,, Germany; 2 Van Andel Institute, Grand Rapids, MI 49503 USA; 3 Max Planck Institute of Immunobiology and Epigenetics, 79108 Freiburg, Germany

**Keywords:** Biomarkers, Obesity, Metabolic disorders

## Abstract

Obesity is a chronic, multifactorial disease which is linked to a number of adverse endocrinological and metabolic conditions. Currently, bariatric surgery is one of the most effective treatments for individuals diagnosed with severe obesity. However, the current indications for bariatric surgery are based on inadequate metrics (i.e., BMI) which do not account for the complexity of the disease, nor the heterogeneity among the patient population. Moreover, there is a lack of understanding with respect to the biological underpinnings that influence successful and sustained weight loss post-bariatric surgery. Studies have implicated age and pre-surgery body weight as two factors that are associated with favorable patient outcomes. Still, there is an urgent medical need to identify other potential factors that could improve the specificity of candidate selection and better inform the treatment plan of patients with obesity. In this report, we present and describe the cohort of the DECON pilot project, a multicenter study which aims to identify predictive biomarkers of successful weight loss after bariatric surgery.

## Introduction

Obesity is defined as excessive fat mass accumulation and it is currently diagnosed by a Body Mass Index (BMI) ≥ 30 kg/m^2^^1^. Obesity has reached epidemic proportions, with an estimated 4 million people dying because of overweight or obesity. In 2016, the World Health Organization (WHO) reported on 650 million people that were considered to be affected by obesity^1^. The prevalence of obesity in individuals ≥ 20 years was 42.4% in the United States between 2017 to 2018^2^. It is associated with comorbidities that negatively affect quality of life and increase mortality, including Type-2 diabetes (T2D), arterial hypertension (aHT), cardiovascular disease (CVD), non-alcoholic fatty liver disease (NAFLD), steatohepatitis (NASH), and malignant tumors^3^. It is also associated with mental health issues including depression and body dissatisfaction^4,5^. Obesity can create workplace performance issues, limit range of motion, decrease productivity and increase absenteeism^6^. Individuals with obesity are typically burdened with higher healthcare and societal costs^7^. Thus, obesity represents a major public health challenge and a considerable socio-economic burden for patients.

Currently, metabolic-bariatric surgery (MBS) is the most effective, long-term treatment option for those who suffer from severe obesity^8^. According to the National Institute of Health (NIH), the candidate selection criteria for surgery should be: (i) BMI ≥ 40 kg/m^2^; (ii) BMI ≥ 35 kg/m^2^ and one or more serious obesity-related health problems; (iii) BMI ≥ 30 kg/m^2^ and obesity-related severe T2D, refractory to medical interventions and lifestyle changes. MBS typically involves gastric volume restriction and/or intestinal bypass to reduce calorie intake. MBS is effective to achieve weight loss, induce partial or complete remission of obesity associated comorbidities, reduce overall and cardiovascular morbidity, incidence rate of malignant tumors, and improve quality of life^8^.

MBS includes distinct procedures, each with indications and contraindications. One of the most common procedures is sleeve gastrectomy (SG), in which nearly 80% of the stomach is resected^9^. The aim of the procedure is to restrict the amount of food or drink that can enter the stomach, reducing caloric intake. The main target of surgical resection in SG is the gastric fundus, a site of abundant ghrelin production^10^. Given the role of ghrelin in stimulating appetite, SG facilitates weight loss and control of blood glucose levels^11^. The second most common procedure is Roux-en-Y gastric bypass (RYGB), in which the proximal portion of the stomach is sectioned off to form a small gastric pouch^12^. This pouch is reattached to the jejunum (alimentary limb) and the remaining larger portion of the stomach, as well as the duodenal and proximal jejunal segments, are bypassed and no longer function in digestion^13^. The biliopancreatic limb is reconnected via anastomosis to the alimentary limb so that bile acids and pancreatic enzymes can enter the small intestine^13^. RYGB physically limits food intake, reduces macromolecule absorption, and enhances satiety^14^.

MBS has a low short-term complication rate. The most frequent long-term complications include gastroesophageal reflux disease (~ 25%), clinically relevant dumping syndrome (~ 7%) and vitamin deficiencies^15^. For a percentage of patients, surgery is unsuccessful. MBS failure has been defined as the inability to lose more than 50% of excess body weight (EBW) or 20% of total body weight (TBW) within the first-year post-operation, or as a weight regain beyond these cut-off values^16^. Between 15 to 35% of patients are nonresponsive to the intervention and suffer from insufficient weight loss (IWL)^17^. Even more problematic, a small subset of patients experiences significant weight regain (WR) and recurrence of associated medical conditions^18–20^. Such relapse is alarming as patients live with both worsening obesity and, potentially, side-effects of surgery.

The causes of heterogeneous treatment response after MBS are not clear. Studies in rodents suggest that surgery reprograms intestinal glucose metabolism^21^ and induces a new metabolic state that is different from the metabolic state induced by dieting alone^22^. Clinical studies have shown associations between MBS failure and patient age, BMI, male sex, parental obesity, T2D, MBS procedure type, early onset of obesity (< 18 years), demographics at the time of surgery, and with immediate post-surgery weight-loss and behavior plans (nutritional/exercise)^16,23^. There is also evidence that MBS has organ-specific effects on RNA expression and DNA-methylation in human patients, including at genes for insulin/insulin-like signaling and intermediate metabolism^24^. The associations defined to date are weak and carry no practical predictive value^25,26^. Likewise, while clinically useful, current criteria used to qualify for MBS neither consider the clinical heterogeneity that exists between individuals with obesity^27,28^, nor do they stratify MBS patients according to overall mortality risk and obesity associated disease burden. They make no mention of the range of presentations including, but not limited to: body morphology, % lean mass vs fat mass, subcutaneous vs visceral fat deposition, degree of insulin resistance, comorbidities that fall above or below the normal range of variability^29^, or metabolic^30^, genetic^31,32^ and epigenetic^33,34^ susceptibilities. Hence, the metabolic and molecular determinants for MBS success or relapse remain largely unknown.

In this report, we introduce the “DECONvolution of obesity sub-groups” (DECON) pilot project cohort study. The cohort comprises female MBS patients with severe obesity, who underwent surgery in 2019, and for whom we collected longitudinal clinical data and the following biological specimens: blood, stool, liver, visceral adipose tissue (VAT) and subcutaneous abdominal adipose tissue (SAT) biopsies. Inclusion criteria of the pilot cohort were intentionally narrow to investigate the feasibility of identifying MBS outcomes, despite the high degree of clinical homogeneity. The samples will be used to generate high-dimensional longitudinal transcriptomic and metabolic datasets. The purpose of this cohort study will be to identify the complex molecular and metabolic sub-groups that distinguish successful and nonresponsive MBS patients and to inform design of a larger future multi-center study aimed at identifying and stratifying surgery response across obesity sub-types and spectrum. It will meet the urgent medical need to identify predictive markers of MBS success, which could guide patient counseling, selection, and lead to improved outcomes.

## Materials and methods

### Ethics approval

This study was performed in line with the principles of the Declaration of Helsinki. Approval was obtained from the Ethik Kommision EK 194/18 and registered at the Deutsches Register für klinische Studien (DRKS) with the registration number DRKS-ID: DRKS00015814. An English version of the study protocol is available at https://drks.de/search/en/trial/DRKS00015814. Date of registration is 09/11/2018. All participants provided written informed consent. All research was performed in accordance with relevant guidelines/regulations. All information were anonymized and images that may identify individuals were not provided in this work.

### Participants

**Figure 1:**
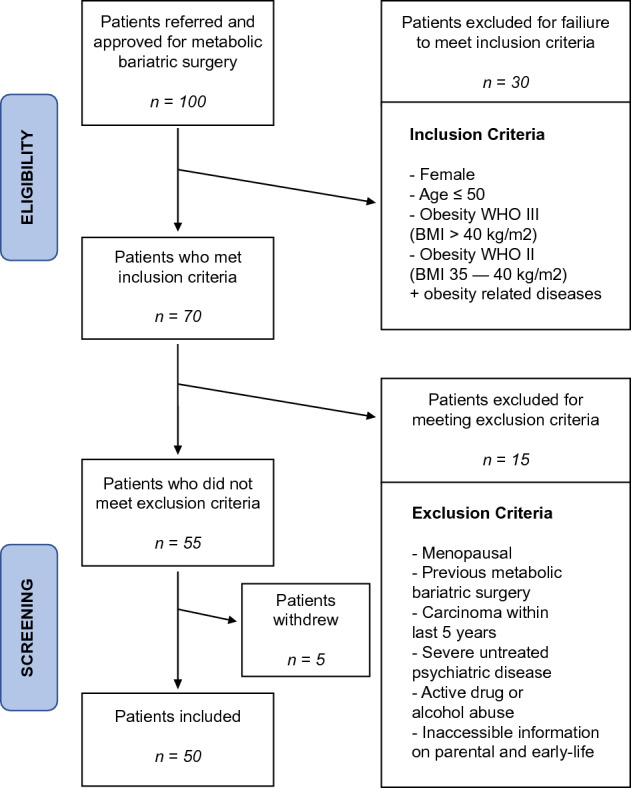
Flowchart and eligibility criteria of participants in the study. Flowchart showing the recruitment steps of the DECON pilot cohort. Inclusion and exclusion criteria are indicated, as well as the number (n) of patients at each step. BMI = body mass index; WHO = world health organization.

Recruitment of the DECON cohort took place in 2019. The target population was adult, pre-menopausal women between 18 and 50 years, with public insurance coverage, and eligible for MBS (see inclusion and exclusion criteria in the following sections, and Fig. 1). The flowchart in Fig. 1 shows the progress of participants through the recruitment of the cohort. The cohort comprised individuals who were referred for MBS to the Medical Center, University of Freiburg (UKF), with a recruitment goal of 50 patients. Potential study participants were first identified through referral (by a primary care physician) and approval (by a specialist) for MBS. 100 patients were identified prospectively. Eligibility was determined through inclusion and exclusion criteria (below). 30 patients were excluded for not meeting the inclusion criteria, leaving 70 patients to advance to the screening stage. 15 patients were excluded for meeting exclusion criteria, and 5 patients withdrew from the study. Thus, 50 patients were included in the final pilot study.

### Inclusion and exclusion criteria

Participant eligibility criteria for this prospective study are reported in Fig. 1. The inclusion criteria were: (1) female sex, (2) younger than 50 years, (3) WHO class III obesity (BMI > 40 kg/m^2^) or (4) WHO class II obesity (BMI > 35 kg/m^2^) with at least one obesity-related disease. The exclusion criteria were: (1) menopausal state, (2) previous MBS, (3) carcinoma diagnosis within last five years, (4) severe and untreated psychiatric disease, (5) active drug or alcohol abuse (except nicotine), (6) lack of accessible information on parental and early life exposures.

### Collection of clinical and anthropometric data

All participants reported to Medical Center, UKF for a preoperative assessment in 2019. Data on demographics, parental predisposition for obesity and T2D, onset of disease, medical history and disease duration, obesity-related disease burden, medication, and smoker status were collected. Participants underwent MBS and a series of follow-up visits at 3, 6, 12 and 24 months. Patients were classified according to the WHO classification and were staged according to the Edmonton Obesity Staging System^35,36^. At each visit, clinical and anthropometric measures were collected, using a Clinical Report Form (CRF) (Supplementary Table 1). Collected measurements included body morphology (e.g., height, weight, BMI, waist and hip circumference, and mid-thigh girth) and a comprehensive metabolic panel. Patients were also asked to provide medical information collected during infancy, early childhood, and adolescence. A summary of anthropometric and clinical measures, and information recorded during preoperative assessment are presented in Tables 1 and 2, respectively.

**Table 1:** Anthropometric and clinical measures of the cohort.

	Overall (N = 50)
Age, years	37.38 ± 7.51
BMI, kg/m^2^	46.47 ± 5.93
WHO obesity classification, N (%)	
Class II (BMI 35—39.9 kg/m^2^)	3 (6%)
Class III (BMI ≥ 40 kg/m^2^)	47 (94%)
WHR	0.88 ± 0.12
WHtR	0.76 ± 0.07
Mid-thigh circumference, cm	72.24 ± 8.26
HbA1c, %	5.73 ± 1.08
HOMA IR, %	1.64 ± 1.14
HOMA B, %	90.26 ± 53.63
Triglycerides, mg/dL	136.80 ± 181.23
Triglyceride level, N (%)	
Normal (< 150 mg/dL)	36 (72%)
Borderline (150—199 mg/dL)	6 (12%)
High (≥ 200 mg/dL)	2 (4%)
Total cholesterol, mg/dL	176.50 ± 32.26
HDL, mg/dL	51.32 ± 21.67
HDL level, N (%)	
Low (< 40 mg/dL)	12 (24%)
Reduced (40—59 mg/dL)	9 (18%)
Normal (≥ 60 mg/dL)	23 (46%)
LDL, mg/dL	118.95 ± 33.79
LDL level, N (%)	
Normal (< 130 mg/dL)	26 (52%)
Borderline (130—159 mg/dL)	16 (32%)
High (≥ 160 mg/dL)	2 (4%)
ALT, U/L	35.04 ± 24.18
ALT level, N (%)	
Normal (< 33 U/L)	32 (64%)
High (≥ 33 U/L)	18 (36%)
AST, U/L	27.02 ± 14.32
AST level, N (%)	
Normal (< 40 U/L)	47 (94%)
High (≥ 40 U/L)	3 (6%)
Uric Acid, mg/dL	5.61 ± 1.14
Uric acid level, (%)	
Normal (< 6 mg/dL)	26 (52%)
High (≥ 6 mg/dL)	18 (36%)
C-reactive protein, mg/L	7.18 ± 6.59
C-reactive protein level, N (%)	
Normal	7 (14%)
High	42 (84%)
Edmonton obesity stage, N (%)	
0	5 (10%)
1	17 (34%)
2	23 (46%)
3	5 (10%)
4	0 (0%)
Surgery type, N (%)	
Sleeve gastrectomy	13 (26%)
Banded sleeve gastrectomy	2 (4%)
Roux-en-Y Gastric bypass	23 (46%)
Banded Roux-en-Y Gastric bypass	12 (24%)

BMI = body mass index; WHR = waist-to-hip ratio; WHtR = waist-to-height ratio; HbA1c = Hemoglobin A1c; HOMA IR = Homeostatic Model Assessment for Insulin Resistance; HOMA B = Homeostatic Model Assessment for beta-cell function; HDL = high-density lipoprotein; LDL = low-density lipoprotein; ALT = alanine transaminase; AST = aspartate aminotransferase.

Table showing the average ± standard deviation of anthropometric and clinical measurements of the patient cohort at the pre-surgery visit. Stratification of the cohorts into indicated groups is reported as the number of individuals (N) and percentage of the whole cohort (%). Also listed are the Edmonton obesity stages and percentages of patients who underwent each surgery type.

**Table 2:** Pre-operation assessment questionnaire data.

	Overall (N = 50)
Sleep apnea, N (%)	
No	46 (92%)
Yes	4 (8%)
Type 2 diabetes, N (%)	
No	36 (72%)
Yes	14 (28%)
Insulin prescription, N (%)	
No	46 (92%)
Yes	4 (8%)
Hypertension, N (%)	
No	37 (74%)
Yes	13 (26%)
Parent(s) w/ obesity, N (%)	
Neither parent	11 (22%)
Yes—mother	13 (26%)
Yes—father	8 (16%)
Both parents	7 (14%)
Parent(s) w/ diabetes, N (%)	
Neither parent	23 (46%)
Yes—mother	10 (20%)
Yes—father	6 (12%)
Both parents	4 (8%)
Polycystic ovarian syndrome, N (%)	
No	47 (94%)
Yes	3 (6%)
Smoker status, N (%)	
Yes	12 (24%)
No, and never have	21 (42%)
No, but have in past	17 (34%)
Pack years (PY), N (%)	5.62 ± 7.60
Antidepressant(s) prescription, N (%)	
No	45 (90%)
Yes	5 (10%)

PY = number of packs of cigarettes smoked per day by the number of years the person has smoked.

Table showing the responses to questions from the pre-surgery visit. Cohort stratification into indicated groups is reported as the number of individuals (N) and percentage of the whole cohort (%).

### Metabolic-bariatric surgery

As standard of care, all study participants received 6 months of dietary counselling and were prescribed a controlled diet, commonly known as liver shrinking diet^37^, for 6 weeks before the date of surgery. Patients were recommended to adhere to foods that are low in carbohydrates and high in proteins and fiber. The purpose of this diet is to drain the liver of glycogen storage, to make surgery technically easier and safer. A secondary effect of the diet was loss of a small amount of body weight before surgery. The dietary schedule was tailored to adipose tissue distribution (visceral versus peripheral/subcutaneous) and additional T2D, patient body weight and lifestyle. If needed, patients received daily protocols to facilitate adherence. Additionally, patients were recommended to begin multivitamin supplementation. All patients underwent laparoscopic surgery in 2019. Patients received either sleeve gastrectomy (commonly considered "restrictive", n = 15) or gastric bypass surgery (commonly considered "metabolic", n = 35). In addition, 14 patients additionally received a silicone band that was placed around the sleeve or the gastric pouch, approximately 4–6 cm distal of the gastroesophageal junction. The band is intended to prevent secondary dilation. Thus, four different types of surgeries were performed: Sleeve Gastrectomy (SG); Banded Sleeve Gastrectomy (BSG); Roux-en-Y Gastric Bypass (RYGB); Banded Roux-en-Y Gastric Bypass (BRYGB). Most of the patients were discharged 3 days after surgery (SD 0.92). Patient follow-up visits at 3-, 6-, 12- and 24-months included anthropometric and metabolic measurements. Body weight loss (i.e., %EWL, %TWL, and percentage of excess BMI loss, %EBMIL), and serum measures of hormonal and metabolic health (e.g., fasting lipids, insulin, glucose, thyroid hormone, sex hormones, and sensitive CRP) were recorded. Homeostatic model assessment for insulin resistance (HOMA-IR) and β-cell function (HOMA-B) scores were calculated for each patient. Any changes to antihypertensive or antidiabetic medications were noted, as were the manifestation of postoperative discomforts and complications (e.g., vomiting, dysphagia, and reflux). Quality of life (QOL) was assessed using the Bariatric Analysis and Outcome Reporting System (BAROS)^38^.

### Patient specimens

Blood, stool, liver, visceral adipose tissue (VAT) and subcutaneous abdominal adipose tissue (SAT) biopsies were collected from all patients prior to or during surgery.

During surgery, roughly 4-5 g of the omental flap and 0.5-2 g of the abdominal subcutaneous compartment were taken and immediately frozen in liquid nitrogen. The frozen adipose samples were later split into two equal samples and stored separately at -80°. Peripheral surgical liver biopsies of the left liver lobe were taken and split on-table. Half of the biopsy was fixed in 4% formaldehyde and sent to the department of clinical pathology for histopathologic evaluation of coincidental pathologies, as well as scoring of NAFLD^39^ and disease activity^40^. All 4 µm thick slides were scored by two pathologists independently and reviewed in the case of divergent scores. The score comprises assessment of steatosis, lobular inflammation, hepatocyte ballooning, and fibrosis. The second half of the liver biopsy was immediately frozen in liquid nitrogen and stored at -80 °C for later analysis.

Plasma and Peripheral Blood Monocluclear Cells (PBMCs) were collected and isolated from EDTA whole blood during the first (i.e., pre-operation) and at each follow-up visit (3, 6, 12 and 24 months). To take plasma, we centrifuged one EDTA tube for 10 min at 4 °C and 3,000 rpm. Supernatant plasma was taken and centrifuged again for 15 min at 4° and 13,000 rpm. Supernatant plasma was then aliquoted and stored at -20 °C overnight and then transitioned to -80 °C the next day. To harvest PBMCs, we used ficoll density gradient centrifugation. After harvesting, PBMCs were suspended in a cold solution of fetal bovine serum (FBS) at a concentration of 0.5 to 1 × 10^7^c/ml. They were incubated at 4 °C for 10 min, and then 10% dimethyl sulfoxide (DMSO) was added. Specimens were stored at -80 °C overnight and then transitioned into a liquid nitrogen freezer.

**Figure 2:**
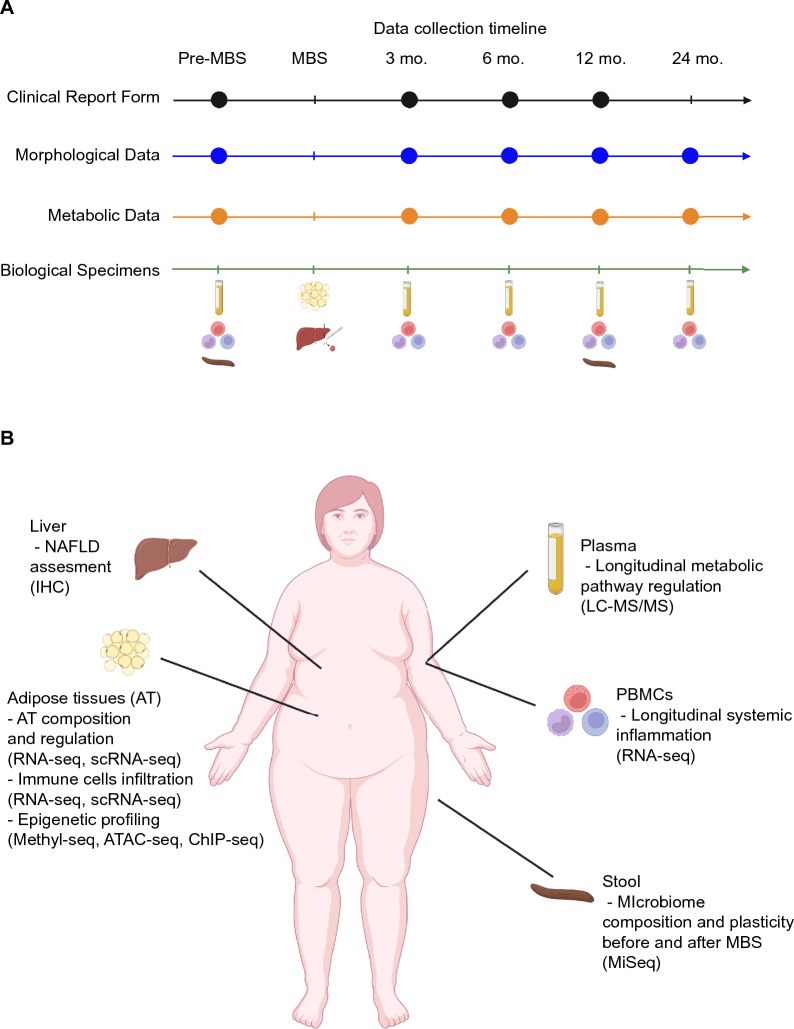
Data and specimens’ collection during the longitudinal study. (**A**) Schematic representation of the longitudinal study and data/specimens collection at the indicated time points. (**B**) Illustration of the biological specimens collected during the study and their possible future applications. Created with BioRender.com. MBS = metabolic-bariatric surgery; mo. = months; IHC = immunohistochemistry; Methyl-seq = DNA methylation sequencing; ATAC-seq = Assay for Transposase-Accessible Chromatin sequencing; ChIP-seq = Chromatin Immunoprecipitation sequencing; LC–MS/MS = liquid chromatography tandem mass spectrometry; RNA-seq = RNA sequencing; MiSeq = microbiome sequencing.

Stool samples were collected in standard fecal sampling tubes by patients at home 1 to 2 days prior to or on the day of the study visit. Patients were instructed to store specimens at -6 °C until departure to the clinic. At arrival, specimens were inspected, aliquoted and then stored at -80°. First time point of collection was ~ 5–7 days prior to surgery when patients visit the clinic for a pre-surgery registration and anesthesiologic exam. The second point of collection was at the 12-month follow-up visit. The biological study samples and time points of collection are illustrated in Fig. 2A.

### Cohort description and longitudinal clustering

**Figure 3:**
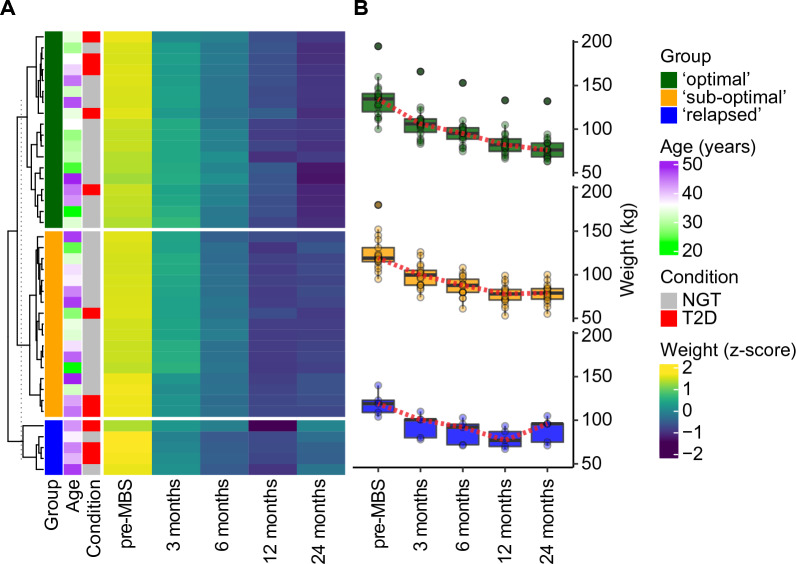
Clustering of patients according to their weight outcome. (**A**) Heatmap visualization of the longitudinal weight measurements during the study. K-means clustering was used to separate patients into 3 main groups of MBS responders. Age and T2D incidence are reported as annotations of the patients. (**B**) Boxplots showing the raw weight measurements trajectories of the identified sub-groups of bariatric surgery responders. Red dotted lines link mean values and highlight the weight trajectories of the three sub-groups. NGT = normal glucose tolerance; T2D = type 2 diabetes; MBS = metabolic-bariatric surgery.

For basic characterization of the pilot cohort, we summarize the distribution and frequency of demographic and clinical traits and risk factors as mean values ± standard deviation (SD) for continuous variables, and proportions for dichotomous variables. In a second step, the cohort was clustered based on longitudinal weight measurements. To have each observation contribute approximately proportionately to the clustering, we performed standardization (Z-score normalization) of the data. The natural logarithm of weight values plus one was calculated, and the log-transformed data were scaled. K-means clustering of the scaled values was performed, using the Ward’s linkage method and Euclidean distance. Heatmap visualization of the clustered data was performed using the package ‘ComplexHeatmap’ v2.12^41^ in an R (v4.2) computing environment. Resulting data clusters were annotated according to patient’s age, BMI, and T2D diagnosis. Raw weight measures were also visualized in addition to standardized values, as comparison. The results are presented in Fig. 3.

## Results

### Anthropometric and clinical description of the cohort

The DECON pilot cohort comprises 50 pre-menopausal females with obesity, who underwent MBS. Baseline anthropometric and clinical traits of the cohort before MBS are reported in Table 1. The average age of the participants was 37.4 years (SD 7.5). The average body weight was 127 kg (SD 19.2), while the average BMI was 46.5 kg/m^2^ (SD 5.93). Most of the patients (94%) were classified as having severe obesity (WHO Class III). Average excess body weight was 58.8 kg.

Waist circumference (WC) and hip circumference (HC) are commonly used to evaluate abdominal adiposity and risk of cardiovascular morbidity and mortality^42^. Before surgery, the average WC was 125 cm (SD 13.4) and the average HC was 147 cm (SD 41.2). Similarly, a waist-to-hip ratio (WHR) greater than 0.85 indicates abdominal obesity in women, and high health risk^43^. A waist-to-height ratio (WHtR) of 0.5 or greater is associated with diabetes risk, and above 0.6 is associated with higher cardiometabolic risk (e.g., myocardial infarction, cardiovascular disease (CVD), and hypertension)^44^. Other studies have found that increased thigh circumference is associated with a decreased incidence of hypertension in individuals suffering from obesity and overweight^45^. In the cohort, 80% of the patients were considered at high risk according to WHR measurements. According to WHtR, instead, 98% and 100% were associated with high cardiometabolic health and diabetes risks. The average girth of the mid-thigh was 72.2 cm (SD 8.2), with 98% showing large measurements.

Blood and metabolic measurements revealed a heterogeneous distribution of metabolic and cardiovascular risk factors. Only 6 patients (12%) showed elevated fasting serum insulin levels. We estimated insulin resistance and β-cell function using the HOMA2-model. The mean pre-surgery HOMA-IR was clearly increased (HOMA-IR = 1.64 ± 1.1 and HOMA-B = 90.3% ± 53.6). Average fasting serum glucose was 114.04 mg/dl (SD 32.3) and HbA1c level was 5.73% (SD 1.1), indicating a general increase in glycemia (66% of participants) and (pre-)diabetic status (32%). 14 patients (28%) were diagnosed with T2D, but only 4 of these were insulin dependent (8%).

We did not observe average deviations from the reference values for serum concentrations of triglycerides (137 ± 181.2 mg/dl), HDL (51.3 ± 21.7 mg/dl), LDL (119 ± 33.8 mg/dl) or total cholesterol (177 ± 32.3 mg/dl). However, 42% of patients showed reduced/low HDL levels, 14% borderline/high cholesterol, 16% borderline/high triglycerides, and 36% borderline/high LDL levels, suggesting increased risk for coronary heart disease, heart attack, and stroke^46^, across the cohort.

Data on disease burden at the beginning of the trial confirmed the association between above measures and increased risk (Table 2). 70% (n = 35) of patients were diagnosed with one or more obesity-associated comorbidity, and 54% (n = 27) with one or more non-obesity associated comorbidity. 78% (n = 39) of the cohort had a mother and/or father who had been diagnosed with overweight or obesity. Of the 50 patients, 26% (n = 13) had been diagnosed with aHT and 24% (n = 12) were on antihypertensive medication(s). 28% (n = 14) of the participants had been diagnosed with T2D. Of these, 29% (n = 4) were insulin dependent. 34% (n = 17) of the patients in the cohort were found to have osteoarthritis. 12% (n = 6) of the patients had been diagnosed with dyslipidemia. 60% (n = 30) of the patient cohort reported a history of smoking. 18% (n = 9) of the patient cohort reported that they were active smokers. 40% (n = 20) of the study participants were prescribed a medication with known and potentially confounding metabolic side effects (thyroid hormone supplements, cortisol, antidepressants). 16% (n = 8) of the patients had lower extremity lipedema.

Of the 50 patients, 30% (n = 15) underwent SG, and 70% (n = 35) underwent RYGB. Of the 15 patients who underwent SG, 2 received a minimizer band (Table 1). Of the 35 patients who underwent RYGB, 12 received a silicone ring.

### Identification of distinct trajectories of weight loss upon metabolic-bariatric surgery

Patient body weights were recorded and tracked over time, starting prior to surgery and at each clinical follow-up. We performed k-means clustering on the longitudinal weight data and identified three distinct groups of patient responses to surgery: (i) successful (‘optimal’) responders, showing continued weight loss with time; (ii) inefficient (‘sub-optimal’) responders, showing stalled weight loss, after 12 months post-intervention; (iii) unsuccessful (‘relapsed’) responders, showing first signs of weight regain from 12 months post-intervention (Fig. 3). According to this clustering, ‘optimal’ and ‘sub-optimal’ responders were equally represented and comprised most of the cohort (‘optimal’ = 45%, ‘sub-optimal’ = 42.5% of the cohort). Consistent with the current literature^17^, a smaller percentage (12.5%) of the cohort relapsed. Relapsed patients were on average older (43 year versus 35 and 38 years for ‘optimal’ and ‘sub-optimal’ responders), and with a higher incidence of T2D (60% versus 28% and 18% in ‘optimal’ and ‘sub-optimal’ responders), in line with previous findings^16^. On a clinical level, TWL in the cohort was above average. Specifically, there are no “poor responders” according to the most common weight loss criterion of TWL 20%. Age at surgery and therefore duration of disease prior to surgery were lower compared to the general MBS population. Average BMI at surgery was also moderately lower compared to the general population in German MBS registries. Within the inclusion criteria, these data indicate that the DECON pilot cohort recapitulates the overall distribution of associated disease and risk factors (e.g. T2D or dyslipidemia) observed in the female patient population of comparable age. They also indicate that the cohort has the potential to characterize at least three major sub-groups of conventional “good responders” to bariatric surgery.

The pre-surgery average BMI in ‘optimal’, ‘sub-optimal’ and ‘relapsed’ patients was 48.18 kg/m^2^, 45.79 kg/m^2^ and 44.80 kg/m^2^, respectively. At the 24-month postoperative visit, the average BMI values decreased to 28.47 kg/m^2^, 28.84 kg/m^2^ and 33.31 kg/m^2^, respectively. At the same time, the average %EWL in ‘optimal’, ‘sub-optimal’ and ‘relapsed’ patients was 87.7%, 82.9% and 60.9%, respectively. The ‘optimal’ outcome of MBS was associated with the highest initial average BMI, somehow in contrast with previous findings, where greater BMI was associated with lower %EWL^47^. Average %TWL was 40.9%, 36.5% and 25.8% and average %EBMIL was 79.1%, 67.8% and 45.4%, respectively. All these metrics show that the three identified sub-groups differ based on these mean therapy endpoints, suggesting the validity of the stratification.

## Discussion

The main goal of the DECON project is to generate a prospective, multi-center cohort with high-quality biologic specimens as a resource for deep interrogation with the ultimate goal of identifying predictive molecular signatures for bariatric surgery outcomes. The DECON project will involve the Department of General and Visceral Surgery, the Institute of Medical Bioinformatics and Systems Medicine and the Workgroup on Vascular Immunology at the Medical Center, University of Freiburg, the Departments of Epigenetics and Metabolism and Nutritional Programming at the Van Andel Research Institute (VAI), and will benefit from the collaboration with the Genomics, Bioinformatics and Biostatistics, and Metabolomics and Bioenergetics facilities at VAI. In this study, we introduce the prospective DECON pilot cohort. Prediction of surgical success on pre-surgical epidemiologic parameters alone, especially on an individual level, remains challenging and vague. All patients in the pilot cohort are Caucasian, women between the ages of 18 and 50 with public insurance coverage. They were sequentially screened and recruited for the study. The pilot cohort is too small to be representative of the entire surgical population. However, it is representative of the female metabolic-bariatric patient population in Germany of comparable age in several respects, e.g., BMI distribution, proportion of patients with mild to severe T2D. The cohort has been carefully characterized on an epidemiologic and clinical level with longitudinal outcome reporting. Despite relatively narrow clinical inclusion criteria and highly standardized procedures, patients show distinct longitudinal responses to surgery. Using the biologic specimens collected during the study we aim at characterizing the participants in terms of molecular, metabolic and microbiome heterogeneity, assessing poorly understood aspects of the effects of bariatric surgery.

Pending funding, we aim to integrate transcriptional profiling of subcutaneous and visceral adipose as well as longitudinal profiling of PBMCs (Fig. 2) and use the resulting datasets to probe for molecular signatures predictive of clinical outcome measures. In addition to this practical translational goal, the datasets will provide deep insight into the phenotypic heterogeneity of the bariatric surgery population with regards to gene expression, post-surgery gene expression dynamics, and therefore provide insight into the regulation and plasticity of the human metabolic condition. We will also be able to gauge the inflammatory state of each patient and investigate the enrichment of inflammatory biomarkers.

While the PBMC samples were collected longitudinally during the study, the liver, VAT and SAT biopsies were obtained during MBS (Fig. 2). These samples will help to elucidate the molecular ‘steady-state’ level of each individual in an unprecedented manner that integrates patient-matched high-dimensional and pathological analyses of distinct adipose depots, liver (including pathology assessment), inflammatory signatures (PBMC transcriptomes), as well as pre- and post-surgery microbiome heterogeneity characteristics and response. Based on the measures of heterogeneity across this pilot population we aim to initiate and expand to a multi-center cohort to define actionable biomarkers for improving diagnosis, stratification, and recommendation for surgery.

Regarding the overarching investigational strategy, a parallel approach can be envisioned, in which: (1) we will perform an outcome-centric analysis, in which we use statistical computation to sort each patient into different surgery responsive groups (e.g., ‘optimal’, ‘sub-optimal’, ‘relapsed’). We will comprehensively characterize these groups and investigate features (i.e., anthropometric, and metabolic traits) that noticeably differentiate the groups from one another; (2) we will perform a heterogeneity-centric analysis, in which we focus on dimensional reduction and graph-based clustering analysis of all data collected prior to surgery. This analysis may provide a stratification of the bariatric surgery population that is more biologically meaningful and less constrained by traditional classification systems. Both approaches will allow us to control both for biological (i.e., age, predispositions) and technical (i.e., batch, surgery procedure type) effects. The matched plasma samples will also be used to map the longitudinal metabolic profiles of patients along the study timeline. We will perform untargeted metabolomic profiling via liquid chromatography tandem mass spectrometry (LC–MS/MS), generating a high-quality dataset that includes thousands of molecules. Furthermore, integration of transcriptional and metabolic profiles will help us in identifying pathways characteristic for the different surgery responses.

Indeed, one evident limitation of the cohort is its relatively small size (50 participants). Our preliminary stratification based on weight trajectories (Fig. 3) suggests that the pilot cohort is recapitulating the overall distribution of MBS outcomes, showing 12.5% of patients suffering from WR. This highlights the potential of this initial pilot cohort. However, one goal of our inter-institutional effort would be to expand the cohort and test our findings in bigger and more complex populations, including other relevant clinical information (i.e., COVID-19 predisposition and disease course).

Ultimately, the integration of deep clinical phenotyping with multi-tissue, -omic and histopathological datasets will provide a rich resource for data-driven characterization of phenotypic and disease heterogeneity in obesity and MBS response.

### Supplementary Information

Supplementary Information.

## Data Availability

The datasets generated during and/or analyzed during the current study are available from the corresponding author on reasonable request.
