# Supplementary material for: The DECON pilot project investigates predictive markers for successful bariatric surgery
Source: Sci Rep. 2023 Aug 17;13:13401. doi: 10.1038/s41598-023-40452-7 (PMC10435485; doi:10.1038/s41598-023-40452-7)
Supplement: Supplementary file 1 — Supplementary Information. [file 41598_2023_40452_MOESM1_ESM.pdf]

Supplementary Table 1. Clinical Report Form (CRF) for the DECON pilot cohort.

Lab values and clinical data collection form, for all the visits of the DECON pilot patients.

CRF

DECONvolution of Obesity

Center for Obesity and Metabolic Surgery
Department of General and Visceral Surgery, University Medical Center Freiburg, University of Freiburg

Patient-ID: |______________|__|__|__|

Patient Initials: |__|__|/|__|__|
Name Surname

Screening

**Patient Info:**	Date of Birth	|__I__|/ 20|__I__| Month Year
	Date of Operation	|__I__|/|__I__|/ 20|__I__|
	Gender	□ m □ w
	Height	________(cm)
	Weight	________(kg)
	BMI	________(kg/m^2)

Inclusion

Patient-ID: |__|__|__|__|

Screening Date: |__I__|/|__I__|/ 20|__I__|
Day Month Year

Inclusion Criteria

The patient is at least 18 years of age and younger than 50 years of age at the time of consenting to participate in the study	□Yes □ No
**BMI 30-35 and severe type 2 diabetes (Metabolic Indication)**<br><br>**or**<br>BMI > 40 kg/m^2<br>**or**<br>BMI ≥ 35-40 kg/m^2 <u>and</u> presence of obesity associated comorbidities such as:<br>○ Type 2 Diabetes<br>○ Arterial Hypertension<br>○ Hyperlipidemia<br>○ Sleep Apnea<br>○ Osteoarthritis<br>○ Obesity Hypoventilation Syndrome<br>○ NAFLD<br>○ NASH<br>○ Idiopathic Intracranial Hypertension<br>○ Gastroesophageal Reflux Disease<br>○ Asthma<br>○ Venous Stasis Disease<br>○ Severe Urinary Incontinence<br>○ Contraindication(s) to other necessary operations due to obesity	□Yes □ No

Signed Consent Form	□ Yes □ No
Female	□ Yes □ No
Caucasian	□ Yes □ No

Exclusion Criteria

Previous bariatric surgery	□ Yes □ No
Carcinoma in the last years	□ Yes □ No
Severe, untreated psychiatric disorder	□ Yes □ No
Drug or alcohol abuse, liver cirrhosis	□ Yes □ No

Date Patient Information and Consent Obtained

|__I__|/|__I__|/ 20|__I__|

Day Month Year

_________________ |__I__|/|__I__|/ 20|__I__| ______________________

Ort Day Month Year Signature (Investigator)

Visit 1 (Pre-surgical evaluation)

Type 2 Diabetes<br>• Year of diagnosis<br>• Metformin<br>• GLP-1 Analogue<br>• Other Oral Antidiabetic Medications<br>• Insulin<br>• Cumulative Insulin Dose	 … ....(Year)<br>□ Yes □ No<br>□ Yes □ No<br>□ Yes □ No<br>□ Yes □ No<br>………………………………..
Arterial Hypertension:<br>• Year of diagnosis<br>• Antihypertensive medication (yes/no)<br>• Number of antihypertensive medications	 … .... (Year)<br>□ Yes □ No<br>………………………………..
Prevalence of maternal or paternal obesity	□ Yes □ No
History of maternal or paternal type 2 diabetes	□ Yes □ No
Lipedema	□ Yes □ No
Lymphedema	□ Yes □ No
Comorbidities (Non-Obesity Related)	□ Yes □ No<br>Which: (with year diagnosed)
Post-Cholecystectomy<br>Post-Ovariectomy<br>Post-Hysterectomy<br>Post-Thyroidectomy	□ Yes □ No<br>□ Yes □ No<br>□ Yes □ No<br>□ Yes □ No
Smoker<br>Pack-Years	□ Yes □ No
Potentially confounding medication:<br><br>Cortisone<br>Thyroid Hormone<br>Lipid-Lowering Drugs<br>Uric Acid-Lowering Drugs<br>Antidepressants	 <br>□ Yes □ No<br>□ Yes □ No<br>□ Yes □ No<br>□ Yes □ No<br>□ Yes □ No

Edmonton Obesity Stage	
Waist Circumference	cm
Hip Circumference	cm
Mid-Thigh Circumference	cm
VAS-Score (Leg Pain)	
Total Score on B.A.R.O.S QoL questionnaire	

Lab values

Blood Test Date: |__I__|/|__I__|/ 20|__I__|
Day Month Year

Parameter	Measurement	Comments<br>**in case of deviations from reference values**
Hemoglobin		
Calcium		
HbA1C Level		
Serum Creatinine		
Serum Albumin		
Total Protein		
C-Reactive Protein		
Iron		
Transferrin		
Folic Acid		
Vitamin B_1		
Vitamin D_3		
Vitamin B_{12}		
Uric Acid		
Total Cholesterol		
LDL		
HDL		
Triglyceride		
ß-HCG*		

* Only women of child bearing age (i.e., non-menopausal)

Operation

Type of surgery:

Sleeve ◻ Yes ◻ No

Banded Sleeve ◻ Yes ◻ No

Gastric Bypass ◻ Yes ◻ No

Biliopancreatic limb length:

Alimentary limb length:

Banded Gastric Bypass ◻ Yes ◻ No

Date of Operation: |__I__|/|__I__|/ 20|__I__|

Day Month Year

Subjective Operation Difficulty Scale (SODS)

1◻ 2◻ 3◻ 4◻ 5◻ 6◻ 7◻ 8◻ 9◻ 10◻

Patient-ID: |__|__|__|__|

Patient-Initials: |__|__| / |__|__|

Name Surname

Sample No. Adipose Tissue: **Subcut.** |__|__|__|__|

Visceral |__|__|__|__|

Sample No. Liver: |__|__|__|__|

Sample No. Blood: |__|__|__|__|

Sample No. Stool: |__|__|__|__|

Visit 2 (Discharge)

Discharge date: |__I__|/|__I__|/ 20|__I__|
Day Month Year

Patient-ID: |__|__|__|__|

Patient-Initials: |__|__| / |__|__|
Name **Surname**

Lab values (2. Postop Day)

Blood Test Date: |__I__|/|__I__|/ 20|__I__|
Day Month Year

**Parameter**	**Measurement**	**Comments**<br>**in case of deviations from reference values**
C-Reactive Protein		

**Postoperative Length of Stay**	
**Surgical Revision**	☐ Yes<br>☐ No
**Endoscopic Treatment**	☐ Yes<br>☐ No
**Drain Placement**	☐ Yes<br>☐ No

**Adverse events (UE)**	Yes ☐<br>No ☐	Yes → Fill out UE Protocol<br><br>No

**Additional Notes:**

Visit 4 (3 months post-surgery)

Date: |__I__|/|__I__|/ 20|__I__|
Day Month Year

Patient-ID: |__|__|__|__|

Patient-Initials: |__|__| / |__|__|
Name **Surname**

Lab values

Blood Test Date: |__I__|/|__I__|/ 20|__I__|
Day Month Year

**Parameter**	**Measurement**	**Comments**<br>**in case of deviations from reference values**
Hemoglobin	__________________g/dl	
Ferritin		
Calcium	_________________mmol/l	
Parathyroid Hormone		
Vitamin D		
Vitamin B12		
HbA1C	_________________%	
Serum Creatinine	__________________mg/dl	
C-Reactive Protein		

Sample No. Blood: |__|__|__|__|

Clinical Evaluation

Weight	kg

Typ 2 Diabetes<br>• Metformin<br>• GLP-1 Analoga<br>• Other Oral Antidiabetic Medication(s)<br>• Insulin<br>• Cumulative Insulin Dose	 □ Yes □ No<br>□ Yes □ No<br>□ Yes □ No<br>□ Yes □ No<br>………………………………..
Arterial Hypertensinon with:<br>• Antihypertensive Medication (yes/no)<br>• Number of Antihypertensive Medications	 □ Yes □ No<br>………………………………..
Vomiting / Regurgitation	No<br>≥ 1/ Week<br>≥ 1/ Month
Dysphagia (degree)	0: Normal foods tolerated<br>1: Some solid foods tolerated<br>2: Only soft foods tolerated<br>3: Only liquid foods tolerated<br>4: Complegte dysphagia
Heartburn / Acid Reflux	No<br>≥ 1/ Week<br>≥ 1/ Month
Smoker<br>Pack-Years	□ Yes □ No
Total Score B.A.R.O.S QoL questionnaire	
Potentially confounding medication:<br>Cortisone<br>Thyroid Hormone<br>Lipid-Lowering Drugs<br>Uric Acid-Lowering Drugs	 □ Yes □ No<br>□ Yes □ No<br>□ Yes □ No<br>□ Yes □ No

Antidepressants	□ Yes □ No

**Dumping Syndrome**	□ Yes<br>□ No
**Endoscopic Treatment (e.g., Dilatation, BARS)**	□ Yes<br>□ No
**Cholecystectomy**	□ Yes<br>□ No
**Bariatric Revision Surgery**	□ Yes<br>□ No

**Adverse events (UE)**	Yes □<br>No □	Yes → Fill out UE Protocol<br><br>No

**Space for Notes:**

Visit 5 (6 months post-surgery)

Date: |__I__|/|__I__|/ 20|__I__|
Day Month Year

Patient-ID: |__|__|__|__|

Patient-Initials: |__|__| / |__|__|
Name **Surname**

Lab values

Blood Test Date: |__I__|/|__I__|/ 20|__I__|
Day Month Year

**Parameter**	**Measurement**	**Comments**<br>**in case of deviations from reference values**
Hemolgobin	__________________g/dl	
Ferritin		
Calcium	_________________mmol/l	
Parathyroid Hormone		
Vitamin D		
Vitamin B12		
HbA1C	_________________%	
Serum Creatinine	__________________mg/dl	
C-Reactive Protein		

Sample No. Blood: |__|__|__|__|

Clinical evaluation

Weight	kg
BMI	kg/m²

Typ 2 Diabetes<br>• First Diagnosed with Diabetes<br>• Metformin<br>• GLP-1 Analogue<br>• Other Oral Antidiabetics<br>• Insulin<br>• Cumulative Insulin Dose	 … ....(Year)<br>□ Yes □ No<br>□ Yes □ No<br>□ Yes □ No<br>□ Yes □ No<br>………………………………..
Arterial Hypertension:<br>• Antihypertensive Medication (yes/no)<br>• Number of Antihypertensive Medications	 □ Yes □ No<br>………………………………..
Vomiting / Regurgitation	No<br>≥ 1/ Week<br>≥ 1/ Month
Dysphagia (degree)	0: Normal foods tolerated<br>1: Some solid foods tolerated<br>2: Only soft foods tolerated<br>3: Only liquid foods tolerated<br>4: Complete dysphagia
Heartburn / Acid Reflux	No<br>≥ 1/ Week<br>≥ 1/ Month
Smoker<br>Packyears	□ Yes □ No
Total Score B.A.R.O.S QoL questionnaire	

Potentially confounding medication:	
Cortisone	□ Yes □ No
Thyroid Hormone	□ Yes □ No
Lipid-Lowering Drugs	□ Yes □ No
Uric Acid-Lowering Drugs	□ Yes □ No

Antidepressants	◻ Yes ◻ No

**Dumping Syndrome**	◻ Yes<br>◻ No
**Endoscopic Treatment (e.g., Dilatation, BARS)**	◻ Yes<br>◻ No
**Cystectomy**	◻ Yes<br>◻ No
**Bariatric Revision Surgery**	◻ Yes<br>◻ No

**Any adverse events (UE)**	Yes ◻<br>No ◻	Yes → Fill out UE Protocol<br><br>No

Space for Notes:

Visit 6 (12 months post-surgery)

Date: |__I__|/|__I__|/ 20|__I__|
Day Month Year

Patient-ID: |__|__|__|__|

Patient-Initials: |__|__| / |__|__|
Name **Surname**

Lab values

Blood Test Date: |__I__|/|__I__|/ 20|__I__|
Day Month Year

Parameter	Measurement	Comments<br>in case of deviations from reference values
Hemoglobin		
Calcium		
HbA1C Level		
Serum Creatinine		
Serum Albumin		
Total Protein		
C-Reactive Protein		
Iron		
Transferrin		
Folic Acid		
Vitamin B_1		
Vitamin D_3		
Vitamin B_{12}		

Uric Acid		
Total Cholesterol		
LDL		
HDL		
Triglyceride		
ß-HCG*		

* Only women of childbearing potential (i.e., non-menopausal)

Clinical evaluation

Hip Circumference	cm
Waist Circumference	cm
Weight	kg
BMI	kg/m^2
Mid-Thigh Circumference	cm
VAS-Score (Leg Pain)	

Type 2 Diabetes<br>• Metformin<br>• GLP-1 Analogue<br>• Other Oral Antidiabetic Medications<br>• Insulin<br>• Cumulative Insulin Dose	 □ Yes □ No<br>□ Yes □ No<br>□ Yes □ No<br>□ Yes □ No<br>………………………………..
Arterial Hypertension:<br>• Antihypertensive Medication (yes/no)<br>• Number of Antihypertensive Medications	 □ Yes □ No<br>………………………………..
Vomiting / Regurgitation	No<br>≥ 1/ Week<br>≥ 1/ Month
Dysphagia (degree)	0: Normal foods tolerated<br>1: Some foods tolerated

	2: Only soft foods tolerated<br>3: Only liquid foods tolerated<br>4: Complete dysphagia
Heartburn / Acid Reflux	No<br>≥ 1/ Week<br>≥ 1/ Month
Smoker<br>Pack-Years	□ Yes □ nein
Potentially confounding medication:<br>Cortisone<br>Thyroid Hormone<br>Lipid-Lowering Drugs<br>Uric Acid Lowering Drugs<br>Antidepressants	 □ Yes □ No<br>□ Yes □ No<br>□ Yes □ No<br>□ Yes □ No<br>□ Yes □ No
Edmonton Obesity Stage	
Total Score B.A.R.O.S QoL questionnaire	
Smoker<br>Pack-Years	□ Yes □ No

**Dumping Syndrome**	□ Yes<br>□ No
**Endoscopic Treatment (e.g., Dilatation, BARS)**	□ Yes<br>□ No
**Cholecystectomy**	□ Yes<br>□ No
**Bariatric Revision Surgery**	□ Yes<br>□ No

**Adverse events (UE)**	Yes ☐<br>No ☐	Yes → Fill out UE Protocol<br><br>No

Sample No. Blood: |__|__|__|__|

Sample No. Stool: |__|__|__|__|

**Space for Notes:**

Final protocol

Date: |__I__|/|__I__|/|__I__| = postOP day No. ____
Day Month Year

Patient-ID: |__|__|__|__|

Patient-Initials: |__|__| / |__|__|
Name Surname

The above mentioned patient left the study by the date mentioned above.

The patient identified above withdrew from the study on the date identified above

**Reason for withdrawal from study**	Wish of patient	Decline to give reason<br><br>Reason given:
	Other reason	Which:

End of study protocol completed by:

______________ |__I__|/|__I__|/ 20|__I__| ______________
Location Day Month Year Signature (Investigator)

Adverse Events (UE)

Patient-ID: |__|__|__|__| Patient-Initials: |__|__| / |__|__|

**UE No.:**	
**UE-Description:**	
**Has the study endpoint been reached?**	□ Yes<br>□ No
**SAE:**	□ Yes<br>□ No

Protocol completed by

______________ |__ı__|/|__ı__|/ 20|__ı__| ____________________

Ort Day Month Year Signature (Investigator)
